# Metformin for the prevention of diabetes among people with HIV and either impaired fasting glucose or impaired glucose tolerance (prediabetes) in Tanzania: a Phase II randomised placebo-controlled trial

**DOI:** 10.1007/s00125-023-05968-7

**Published:** 2023-07-18

**Authors:** Anupam Garrib, Sokoine Kivuyo, Katie Bates, Kaushik Ramaiya, Duolao Wang, Edna Majaliwa, Rehema Simbauranga, Godbless Charles, Erik van Widenfelt, Huanyan Luo, Uazman Alam, Moffat J. Nyirenda, Shabbar Jaffar, Sayoki Mfinanga, on behalf of the META trial team

**Affiliations:** 1 UCL Institute for Global Health, University College London, London, UK; 2 Department of Clinical Sciences, Liverpool School of Tropical Medicine, Liverpool, UK; 3 Muhimbili Medical Research Centre, National Institute for Medical Research, Dar es Salaam, Tanzania; 4 Institute of Medical Statistics and Informatics, Medical University Innsbruck, Innsbruck, Austria; 5 Shree Hindu Mandal Hospital, Dar es Salaam, Tanzania; 6 Department of Cardiovascular and Metabolic Medicine, Institute of Life Course and Medical Sciences, University of Liverpool, Liverpool, UK; 7 Liverpool University NHS Hospital Foundation Trust, Liverpool, UK; 8 Department of Diabetes, Endocrinology and Gastroenterology, Faculty of Biology, Medicine and Health, University of Manchester, Manchester, UK; 9 Department of Non-communicable Disease Epidemiology, London School of Hygiene and Tropical Medicine (LSHTM), London, UK; 10 NCD Theme, MRC/UVRI & LSHTM Uganda Research Unit, Entebbe, Uganda

**Keywords:** HIV, Metformin, Prediabetes, Prevention, Tanzania

## Abstract

**Aims/hypothesis:**

In sub-Saharan Africa (SSA), 5% of adults are living with type 2 diabetes and this is rising sharply, with a greater increase among people with HIV. Evidence on the efficacy of prevention strategies in this cohort is scarce. We conducted a Phase II double-blind placebo-controlled trial that aimed to determine the impact of metformin on blood glucose levels among people with prediabetes (defined as impaired fasting glucose [IFG] and/or impaired glucose tolerance [IGT]) and HIV in SSA.

**Methods:**

Adults (≥18 years old) who were stable in HIV care and found to have prediabetes (IFG and/or IGT) and who were attending hospitals in Dar es Salaam, Tanzania, were randomised to receive sustained-release metformin, 2000 mg daily, or matching placebo between 4 November 2019 and 21 July 2020. Randomisation used permuted blocks. Allocation was concealed in the trial database and made visible only to the Chief Pharmacist after consent was taken. All participants, research and clinical staff remained blinded to the allocation. Participants were provided with information on diet and lifestyle and had access to various health information following the start of the coronavirus disease 2019 (COVID-19) pandemic. Participants were followed up for 12 months. The primary outcome measure was capillary blood glucose measured 2 h following a 75 g glucose load. Analyses were by intention-to-treat.

**Results:**

In total, 364 participants (182 in each arm) were randomised to the metformin or placebo group. At enrolment, in the metformin and placebo arms, mean fasting glucose was 6.37 mmol/l (95% CI 6.23, 6.50) and 6.26 mmol/l (95% CI 6.15, 6.36), respectively, and mean 2 h glucose levels following a 75 g oral glucose load were 8.39 mmol/l (95% CI 8.22, 8.56) and 8.24 mmol/l (95% CI 8.07, 8.41), respectively. At the final assessment at 12 months, 145/182 (79.7%) individuals randomised to metformin compared with 158/182 (86.8%) randomised to placebo indicated that they had taken >95% of their medicines in the previous 28 days (*p*=0.068). At this visit, in the metformin and placebo arms, mean fasting glucose levels were 6.17 mmol/l (95% CI 6.03, 6.30) and 6.30 mmol/l (95% CI 6.18, 6.42), respectively, and mean 2 h glucose levels following a 75 g oral glucose load were 7.88 mmol/l (95% CI 7.65, 8.12) and 7.71 mmol/l (95% CI 7.49, 7.94), respectively. Using a linear mixed model controlling for respective baseline values, the mean difference between the metformin and placebo group (metformin–placebo) was −0.08 mmol/l (95% CI −0.37, 0.20) for fasting glucose and 0.20 mmol/l (95% CI −0.17, 0.58) for glucose levels 2 h post a 75 g glucose load. Weight was significantly lower in the metformin arm than in the placebo arm: using the linear mixed model adjusting for baseline values, the mean difference in weight was −1.47 kg (95% CI −2.58, −0.35). In total, 16/182 (8.8%) individuals had a serious adverse event (Grade 3 or Grade 4 in the Division of Acquired Immunodeficiency Syndrome [DAIDS] adverse event grading table) or died in the metformin arm compared with 18/182 (9.9%) in the placebo arm; these events were either unrelated to or unlikely to be related to the study drugs.

**Conclusions/interpretation:**

Blood glucose decreased over time in both the metformin and placebo arms during the trial but did not differ significantly between the arms at 12 months of follow up. Metformin therapy was found to be safe for use in individuals with HIV and prediabetes. A larger trial with longer follow up is needed to establish if metformin can be safely used for the prevention of diabetes in people who have HIV.

**Trial registration:**

The trial is registered on the International Standard Randomised Controlled Trial Number (ISRCTN) registry (www.isrctn.com/), registration number: ISCRTN76157257.

**Funding:**

This research was funded by the National Institute for Health Research using UK aid from the UK Government to support global health research.

**Graphical Abstract:**

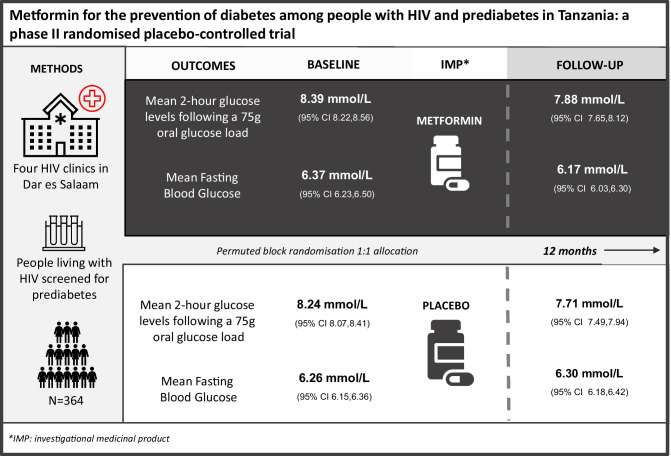


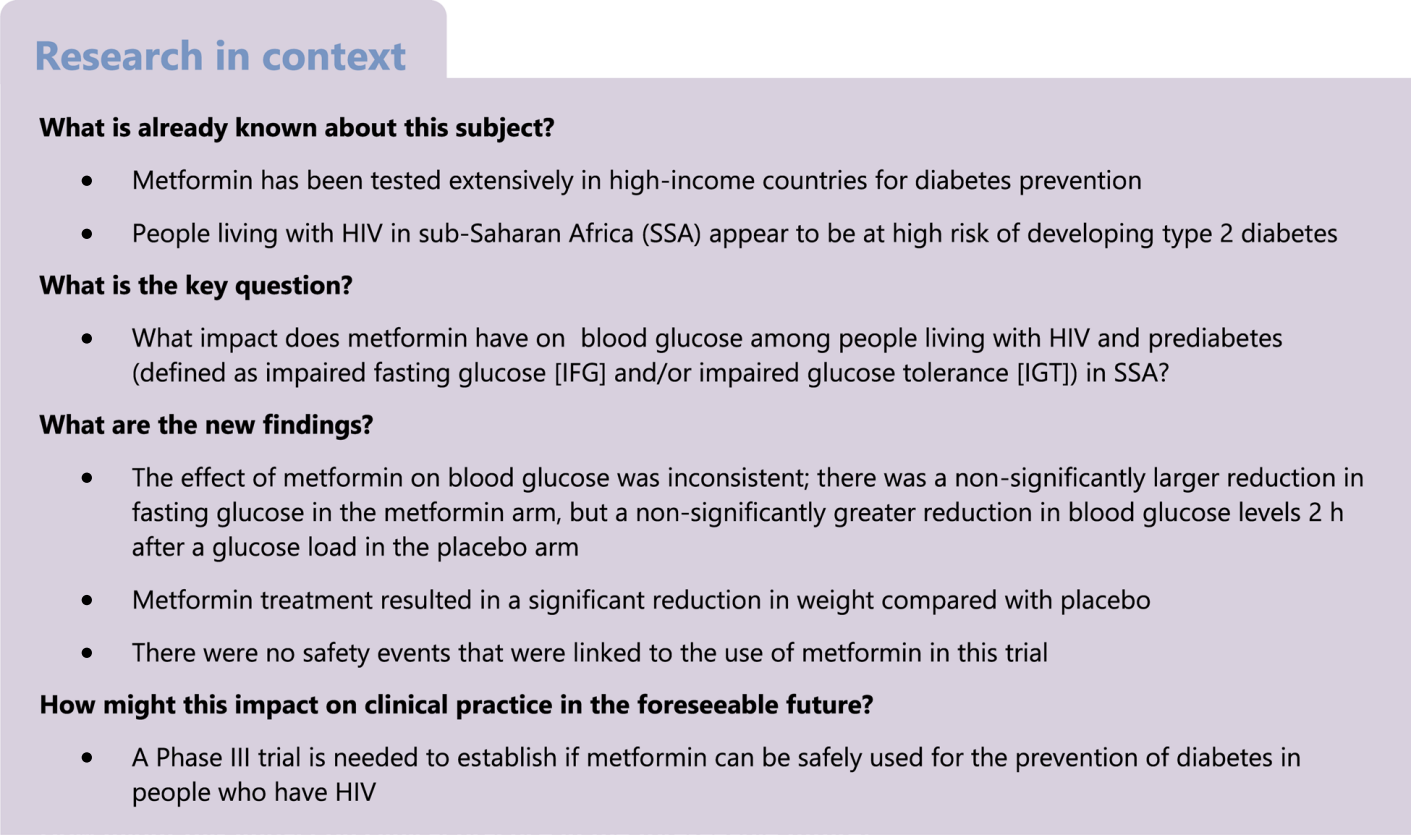


## Introduction

The rapidly rising prevalence of type 2 diabetes mellitus is a global public health threat. In sub-Saharan Africa (SSA), an estimated 5% of adults are living with type 2 diabetes [1] and health service provision is limited, with few individuals with type 2 diabetes thought to be under regular care [2, 3].

Evidence on the efficacy of type 2 diabetes prevention strategies from SSA is scarce. People with blood glucose levels that fall just below the diagnostic level for diabetes are considered to have ‘prediabetes’. Prediabetes comprises heterogeneous states of impaired fasting glucose (IFG), impaired glucose tolerance (IGT) or, in high-income settings, elevated HbA_1c_ [4]. Each of these conditions on its own or in combination may have different underlying pathophysiologies and there is no consensus on how prediabetes should be defined. Nonetheless, people with prediabetes defined according to any of these conditions have a high risk of progression to type 2 diabetes [4]. There are two primary interventional approaches to type 2 diabetes prevention: (1) intensive, structured lifestyle interventions to improve diet and increase physical activity; or (2) pharmacological therapy. These interventions have been evaluated primarily in high-income countries, including Finland [5], the USA [6] and China [7, 8]. Both approaches have been shown to be effective in reducing progression to type 2 diabetes by between 30% and 50%, with greater effects seen with intensive lifestyle interventions than with pharmacotherapy [6, 9–12]. However, intensive lifestyle interventions are time intensive, require skilled healthcare workers (of which there are not many) and would be costly to implement in most settings in SSA.

In randomised trials, delays in progression to type 2 diabetes have been demonstrated with pharmacological agents, including acarbose [13], rosiglitazone [14], pioglitazone [15] and metformin [16–18], when compared with placebo. Of these agents, metformin has been in clinical use for decades as the first-line treatment for type 2 diabetes and has been tested extensively in prediabetes. In a US study (the Diabetes Prevention Program/Diabetes Prevention Programme Outcomes Study [3]), open-label follow up of the study cohort for 15 years suggested that a substantial beneficial effect of metformin in preventing progression to type 2 diabetes was sustained over time [19]. However, metformin does not appear to confer any additional benefit over and above intensive diet and exercise interventions [20].

The prevalence of prediabetes among people with HIV has been reported as being between 18% and 26% in SSA, although these estimates are from small studies [21, 22] and dependent on the population studied and the markers of glycaemia used. Various studies have suggested that the risk of type 2 diabetes is higher among people with HIV [23–25] and this is thought to be attributed to inflammation caused by HIV and metabolic changes caused by antiretroviral therapy (ART) [23, 26, 27]. However, whether the risk of developing type 2 diabetes is elevated among people living with HIV who are virally suppressed is not known.

Dolutegravir is a HIV-integrase inhibitor that, since 2019, has been part of the first-line therapy for HIV in Tanzania [28]. Dolutegravir is, however, known to cause an increase in body weight and increased risk of hyperglycaemia [29, 30]. Whilst there is evidence that dolutegravir causes a decrease in the renal elimination of metformin, there is little evidence of the impact of this on blood glucose or safety of metformin when used together with dolutegravir [31, 32]. It is recommended that a reduction in metformin dose is considered if these drugs are taken together, and that metformin should be used with caution in people with moderate renal impairment due to an increased risk of lactic acidosis.

No trials have been conducted in SSA to evaluate the efficacy of metformin for the prevention of type 2 diabetes. A recent open-label trial conducted among people with HIV and prediabetes in Thailand suggested that metformin may improve glycaemic control compared with no therapy, but this study was small, with just 37 participants in each trial arm [33]. Thus, we conducted a Phase II randomised placebo-controlled trial of metformin vs placebo in people living with HIV and prediabetes in Tanzania.

## Methods

### Trial design and interventions

The Metformin Treatment for Africa (META) trial was a Phase II randomised placebo-controlled trial conducted among people with prediabetes and HIV who were taking ART. Neither the participants or the healthcare staff or the researchers were aware of participant allocation (i.e. the study was double-blinded). The trial was designed to evaluate the effects of metformin on blood glucose and to evaluate drug safety over a 12 month duration. The primary outcome measure was blood glucose levels measured 2 h following a 75 g glucose load as this was felt to provide a more consistently reproducible measure than fasting glucose. Secondary outcome measures included drug safety. Adverse events were graded using the Division of Acquired Immunodeficiency Syndrome (DAIDS) criteria and events Grade 3 or greater were reported as serious adverse events (SAEs) [34].

#### Interventions

Participants were asked to take 2000 mg extended-release metformin hydrochloride, dispensed in 500 mg tablets. Placebos were identical in shape, size and appearance. All trial drugs were provided by Merck Healthcare, Darmstadt, Germany. Participants were asked to take four tablets in the evening after food. They were provided information about possible adverse effects. Participants in both arms also received brief structured advice on diet and lifestyle and on medicine adherence at every visit. Adherence was measured using a visual adherence score and by pill counts.

#### Randomisation and follow up

Eligible participants were randomised to the intervention arm or control arm using permuted block randomisation with varying block sizes of 4, 6 and 8 people chosen at random using the SAS software (SAS version 9.4, SAS Institute, Cary, NC, USA.). Each participant had a unique randomisation code. The randomisation list was held in the Electronic Data Capture database (https://github.com/meta-trial/meta-edc, accessed 23 May 2023) and participants were allocated sequentially to the next available code by research study physicians (GC, RS, EM and F. Pratap [META trial team member; National Institute for Medical Research, Dar es Salaam, Tanzania]), with cross-checks conducted by one of the META trial team pharmacists (trial pharmacists are listed in the Appendix).

The randomisation code was written and tested by a senior statistician (DW) and the final list was generated by an independent trial statistician (T. Chen, Liverpool School of Tropical Medicine [LSTM], Liverpool, UK), who maintained the randomisation codes. A copy of the participant allocation list was held by the META trial team’s Chief Pharmacist and his assistant, I. Tarimo (both Muhimbili National Hospital, Dar es Salaam, Tanzania). All research and clinical staff remained blinded to the allocation code.

Participants were given drug supplies to last them until their next visit plus 6 days’ extra supply in case they were late returning to the clinic. The scheduled visits occurred at 2 weeks and at 1, 3, 6, 9 and 12 months after baseline. Laboratory blood tests of renal and liver function were conducted at 3-monthly intervals. In addition, participants with abnormal baseline laboratory results had unscheduled visits for clinical review and additional tests were immediately conducted to assess potential relatedness with investigational drugs.

#### Sample size

We had no prior data from SSA. We assumed that the mean (SD) glucose level 2 h following a 75 g oral glucose load would be 10.0 (1.5) mmol/l at 12 months in the placebo arm. We calculated that a study with 160 people per arm would have 85% power to detect a difference in blood glucose levels at 2 h post a 75 g glucose load from 10 mmol/l down to 9.5 mmol/l in the metformin arm (i.e. an absolute difference of 0.5 mmol/l between the two arms) and enroled 182 people per arm to allow for losses to follow-up.

#### Trial setting and population

The trial was conducted in the HIV clinics of four hospitals in Dar es Salaam, of which three were public hospitals (Amana, Mwananyamala and Temeke Hospitals) and one was a not-for-profit hospital (Shree Hindu Mandal Hospital). These hospitals serve largely urban populations.

There is no consensus on how to define prediabetes [35]. Evidence of the effects of metformin among people with HIV is scarce, particularly in settings in SSA. We therefore took a cautious approach and biased the eligibility criteria to individuals who were at high risk of developing type 2 diabetes (i.e. they were obese (BMI >30 kg/m^2^), or not obese (BMI ≤30 kg/m^2^) but with higher levels of IFG and/or IGT [refer to inclusion criteria below]). This was intended as a safety precaution to reduce exposure to metformin among individuals who were less likely to progress to type 2 diabetes in the short-to-medium term.

Individuals were invited for screening if they were aged 18 years or older, on ART, regularly attending their HIV clinic for at least 6 months, were not pregnant, did not have a history or clinical evidence of acute metabolic disorders, or kidney, liver or heart disease, and had no contraindication to metformin. Two methods were used to screen individuals to assess their eligibility: (1) purposeful sampling, whereby individuals known or suspected to have prediabetes or individuals with a high BMI (>30 kg/m^2^) were invited for screening; and (2) systematic sampling from the list of individuals expected to attend the clinic on the following day. Potentially eligible individuals were invited to a follow-up appointment for blood glucose level screening via an OGTT. The potential participants were advised to adhere to their usual diet, avoid vigorous physical activity prior to the scheduled clinic visit, and attend the clinic in the morning after an overnight fast of a minimum of 8 h.

Individuals were considered eligible if they had: (1) BMI >30 kg/m^2^ combined with either IFG (6.1–6.9 mmol/l) and/or IGT 2 h following a glucose load (blood glucose: 7.0–11.1 mmol/l); or (2) BMI ≤30 kg/m^2^ combined with either elevated fasting glucose (6.3–6.9 mmol/l) and/or elevated glucose levels 2 h after a glucose load (9.0–11.1 mmol/l). Eligible individuals who had baseline eGFR <45ml/min per 1.73 m^2^ were not enroled into the study. Ethnicity data was collected by participant self-report for the calculation of eGFR.

### Blood/urine sampling and data collection

On arrival at the clinic, a morning blood sample was collected via venepuncture by a research nurse, and fasting blood glucose was measured. Participants were given a 75 g anhydrous glucose solution dissolved in 300 ml non-carbonated water (Rapilose OGTT solution; Galen, Craigavon, UK) and a 2 h post glucose-load capillary blood glucose measurement was taken via finger prick. To determine glucose, a point-of-care test was performed (HemoCue Glucose 201 RT; Hemocue, Ängelholm, Sweden) immediately following venepuncture and finger prick. Further screening investigations included HbA_1c_ analysis (HemoCue HbA_1c_ 501 system), a urine pregnancy test (Laborex, Zhejiang Orient Gene Biotech, Huzhou, China) and urine testing for ketones, protein and glucose (Cybow, DFI, Gimhae, Republic of Korea). In addition, a malaria rapid diagnostic test (a rapid malaria point-of-care antigen test) was conducted (Malaria Combo pf/PAN [HRP2/pLDH] Ag; CareStart, Somerset, NJ, USA).

Venous blood samples were collected every 3 months from enroled participants on arrival to the clinic. Full blood count (FBC), and analysis of total cholesterol, LDL-cholesterol (LDL-C), HDL-cholesterol (HDL-C) and triglyceride levels were conducted at baseline and at the 12 month visit only. For the FBC, blood samples were in EDTA tubes and for biochemical analyses, blood samples were in plain tubes; these samples were transported to a centralised laboratory for processing within 2–3 h of procurement (the mean time between collection and processing was 11 h). The Architect C4100 analyser (Abbott, IL, USA) was used for biochemical analysis, whilst the CELL-DYN 3700 and CELL-DYN Ruby analysers (both Abbott) were used for haematological (FBC) analysis.

Participants with suspected clinical conditions and/or abnormal laboratory markers identified through screening or enrolment procedures were referred to the appropriate clinics within the health facility. Any participants with raised BP, above 180/120 mmHg, were referred to the emergency department for immediate care. Participants in the trial who developed type 2 diabetes were referred to the diabetes service within the health facility. Adverse events were graded using the DAIDS criteria, and events that were Grade 3 or greater were reported as SAEs [34].

Anthropometric measurements included height, weight, and waist and hip circumference, all of which were measured following removal of shoes and outer clothing. Height was measured to the nearest 1 cm using calibrated stadiometers (Seca, Hamburg, Germany) and weight to the nearest 1 kg. using calibrated Seca scales. Flexible tape measures were used to measure waist and hip circumference. The WHO definitions of threshold values were used for classifying BMI, waist circumference and waist:hip ratio [36]. After 10 min of rest, three seated BP measurements were collected on the left arm (these were collected on the right arm in those with conditions that precluded the use of the right arm), using portable sphygmomanometers (M6 Comfort [HEM-7321-E]; OMRON Healthcare, Hoofddorp, the Netherlands), with 5 min of rest in between each measurement. We used the mean of the last two BP readings. Sex (male or female) was determined by participant self-report; no one reported a different sex to the sex assigned at birth.

### Timeline and disruption due to the coronavirus disease 2019 pandemic

Recruitment began on 4 November 2019 and the last participant was enroled on 21 July 2020. Follow up of participants ended 12 months later, as planned. The first confirmed cases of coronavirus disease 2019 (COVID-19) in Tanzania occurred in mid-March 2020 and caused severe disruption [37, 38].In Tanzania, health services reduced the frequency of hospital appointments for individuals with HIV and instead engaged with patients in the community and by phone. Communities also had increased health information on reducing their risk of illness from COVID-19. It was established early that dysglycaemia and obesity were risk factors for severe illness from COVID-19 [39, 40].

We maintained the schedule of appointments following initial meetings with participants. On 17 April 2020, Amana Hospital, our busiest recruitment site, was closed to non-COVID-19 patients, with a 24 h notice period. Trial participants were transferred to Mwananyamala Hospital, which had not previously been a trial site. On 28 August 2020, Amana Hospital was re-opened to all patients and trial participants were transferred back as this was their local health facility.

### Ethics

The protocol was approved by the LSTM Research ethics committee (reference: 17-078), by the National Institute for Medical Research (NIMR)/Ministry of Health Ethics Committee (reference: NIMR/HQ/R.8a/Vol.IX/2916), by the Tanzania National Research Ethics Committee and by the Tanzania Medicine and Devices Authority. All participants provided written informed consent to participate in the study. This trial is registered with the International Standard Randomised Controlled Trial Number (ISRCTN) registry (www.isrctn.com/), registration number ISRCTN76157257.

### Statistical methods

Primary analyses were by intention-to-treat and included participants who were randomised and had at least one valid measurement of primary or secondary outcomes. A linear mixed model was employed for the primary endpoint analysis. The linear mixed model included treatment (metformin vs placebo), visit (midpoint and study end [i.e. approximately 6 and 12 months, respectively]), and the interaction between treatment and visit as fixed effects, controlling for baseline blood glucose values at 2 h post 75 g glucose load, and including a participant level random intercept. Restricted maximum likelihood estimation was used to fit the linear mixed model, assuming an unstructured covariance matrix. The Satterthwaite approximation for the degrees of freedom was used. This model was used to determine the difference in means of glucose levels 2 h post 75 g glucose load (and two-sided 95% CI) between the two treatment arms at midpoint and endpoint. A two-sided *p* value of <0.05 was used to define statistical significance. Fixed-effects parameters were tested with the Wald test. Missing data were treated as missing at random in the mixed model analysis and no imputation of the primary endpoint was made. To assess the sensitivity of the result to this assumption that missing data were missing at random, the multiple imputation and the last observation carried forward strategies were used to compute missing primary endpoints. In addition, a covariate-adjusted model, which incorporated pre-specified baseline covariates (site, age, sex, fasting plasma glucose, 2 h postprandial blood glucose levels, BMI and hypertension) into the above linear mixed model was used to analyse the primary endpoint. Subgroup analyses were performed on the seven following pre-specified covariates at baseline: site, fasting plasma glucose, blood glucose levels at 2 h following a 75 g glucose load, age, sex, BMI and hypertension.

Continuous secondary outcomes were analysed in a similar way as the primary endpoint. For the analysis of binary secondary outcomes, a generalised linear mixed model was employed with treatment, visit, and interaction between treatment and visit as fixed effects, controlling for baseline values of each respective outcome, and including a participant level random intercept. For blood lipids, which were only measured at baseline and 12 month follow-up, estimates were derived using linear regression models controlling for baseline values. The odds ratio between the two treatment arms at each visit, together with 95% CI, was derived from the generalised mixed model. The generalised linear model was used to analyse secondary outcomes with a single follow-up measurement, controlling for baseline values. The occurrence of diabetes was summarised using the number of events and incidence rate by treatment group and analysed using a generalised linear model with Poisson distribution and log link function and with treatment as a fixed effect, to generate incidence rate ratios (IRRs) and 95% CIs. Thresholds for diabetes were defined using the respective thresholds for fasting blood glucose (≥7.0 mmol/l), 2 h postprandial blood glucose level (≥11.1 mmol/l) and HbA_1c_ ≥48 mmol/mol (≥6.5%). The *χ*^2^ test was used to compare proportion of participants with an SAE at each scheduled study visit.

Analyses were performed using SAS software (version 9.4; SAS Institute, Cary, NC, USA). A *p* value <0.05 (two-sided) was considered statistically significant.

## Results

**Fig. 1:**
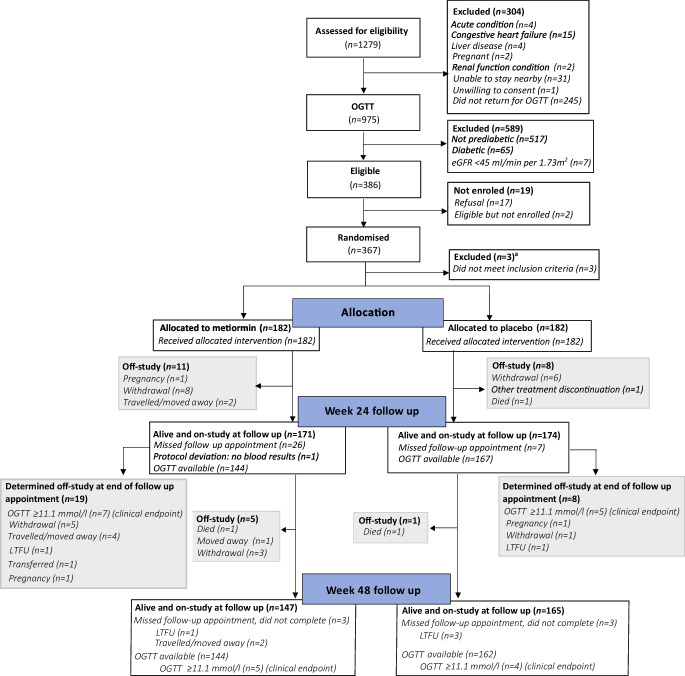
CONSORT flow diagram. ^a^Reason for exclusion of *n*=3 individuals between randomisation and allocation: *n*=2 excluded because, although they met the inclusion criteria based on fasting glucose, glucose values during the OGTT were in the diabetic range and these individuals were incorrectly randomised; *n*=1 excluded owing to error in data entry leading to participant being incorrectly randomised (no investigational medicinal product was dispensed). LTFU, lost to follow-up

In total, 1279 individuals with HIV were invited for screening. Of these, 364 had raised glycaemia and met the enrolment criteria; these individuals were subsequently randomised (Fig. 1). The proportions of individuals that were lost to follow-up, withdrew consent or said that they were moving away were higher in the metformin arm than in the placebo arm (27/182 vs 11/182; *p*=0.006).

Table 1 shows the baseline characteristics, which were well balanced between the two arms. The majority of participants were women (299/364 individuals [82%]) and 222/364 individuals (61.0%) had a BMI ≥30 kg/m^2^. The median age of the cohort was 47 years (range: 25–74 years) and 105/364 individuals (28.8%) had raised total cholesterol. The mean (SD) fasting glucose was 6.3 (0.8) mmol/l, mean blood glucose level at 2 h following a 75 g glucose load was 8.3 (1.2) mmol/l and 110/363 individuals (30.3%) had an HbA_1c_ of 42 mmol/mol (6.0%) or more. In the metformin arm, 166/182 individuals (91.2%) were on an antiretroviral regimen containing dolutegravir compared with 170/182 individuals (93.4%) in the placebo arm. For these 336 individuals, mean BMI (95% CI) was 31.6 kg/m^2^ (30.9, 32.3), whilst among those not on a dolutegravir-based regimen (*n*=28), mean BMI was 29.2 kg/m^2^ (26.8, 31.5). Almost half of the study participants had raised BP (systolic BP >140 mmHg and/or diastolic BP >90 mmHg) and HIV viral suppression exceeded 90%.

**Table 1:** Baseline sociodemographic and clinical indicators according to trial arm

Characteristic	Metformin	Placebo
Participants, (*n*)	182	182
Women, *n* (%)	149 (81.9)	150 (82.4)
Age (years), median (range)	47.0 (28.0–72.0)	46.0 (25.0–74.0)
Duration on ART (months), median (range)	84.4 (7.0–181.1)	86.5 (6.2–176.9)
Current antiretroviral regimen, *n* (%)		
TDF, 3TC, DTG	163 (89.6)	167 (91.8)
Other DTG regimen	3 (1.6)	3 (1.6)
Non-DTG regimen	16 (8.8)	12 (6.6)
Plasma viral load^a^		
Time since last tested (months), median (range)	6.4 (0.0–70.0)	7.2 (0.6–104.2)
>100 copies/ml, *n* (%)	13 (7.3)	11 (6.1)
Weight (kg)		
Median (range)	78.0 (40.0–121.0)	80.0 (44.0–135.0)
Mean (SD)	76.80 (15.76)	81.07 (17.85)
BMI (kg/m^2^)		
Median (range)	30.4 (18.6–45.5)	31.5 (17.6–54.8)
<25, *n* (%)	36 (19.8)	24 (13.2)
25–29.9, *n* (%)	48 (26.4)	34 (18.7)
≥30, *n* (%)	98 (53.8)	124 (68.1)
BP ≥140/90 mmHg, *n* (%)	83 (45.6)	82 (45.1)
Haemoglobin <130.0 g/l in men or <120.0 g/l in women, *n* (%)^b^	62 (35.2)	59 (33.9)
LDL-C (mmol/l), median (range)	3.1 (0.1–7.8)	3.3 (0.1–7.8)^c^
HDL-C (mmol/l), median (range)	1.2 (0.1–2.5)	1.2 (0.3–6.1)
Total cholesterol (mmol/l), median (range)	4.6 (1.3–7.6)	4.7 (2.7–9.2)^d^
Triglycerides (mmol/l), median (range)	1.0 (0.3–3.4)	1.0 (0.3–6.0)
Time fasted (hours), median (IQR)	12.0 (11.0–12.5)	12.0 (11.0–12.8)
2 h post OGTT blood glucose (mmol/l)^e^, mean (95% CI)	8.39 (8.22, 8.56)	8.24 (8.07, 8.41)
Fasting glucose (mmol/l), mean (95% CI)	6.37 (6.23, 6.50)	6.26 (6.15, 6.36)
HbA_1c_ (mmol/mol), mean (95% CI)	39.90 (38.40, 41.41)^d^	38.07 (36.68, 39.47)
HbA_1c_ (%), mean (95% CI)	5.80 (5.66, 5.94)^d^	5.63 (5.51, 5.76)
Glucose levels, *n* (%)		
Fasting <6.1 mmol/l and after glucose load <7.8 mmol/l	20 (11.0)	26 (14.3)
Fasting <6.1 mmol/l and after glucose load 7.8–11.0 mmol/l	32 (17.6)	31 (17.0)
Fasting 6.1–6.9 mmol/l and after glucose load <7.8 mmol/l	48 (26.4)	48 (26.4)
Fasting 6.1–6.9 mmol/l and after glucose load 7.8–11.0 mmol/l	56 (30.8)	53 (29.1)
Fasting ≥7.0 mmol/l and after glucose load <7.8 mmol/l	3 (1.6)	1 (0.5)
Fasting ≥7.0 mmol/l and after glucose load 7.8–11.0 mmol/l	23 (12.6)	23 (12.6)

^a^Missing data for *n*=3 in metformin arm and *n*=1 in placebo arm

^b^Missing data for *n*=6 in metformin arm and *n*=8 in placebo arm

^c^Missing data for *n*=1 (baseline LDL-cholesterol was out of the range of the assay)

^d^Missing baseline data for *n*=1

^e^2 h blood glucose following a 75 g glucose load

DTG, dolutegravir; HDL-C, HDL-cholesterol; LDL-C, LDL-cholesterol; 3TC, lamivudine; TDF, tenofovir disoproxil fumarate

The follow-up duration was similar in both arms (Table 2). Overall, 277/364 participants (76.1%) completed the study and had glycaemia tests as scheduled or within 6 days of their final appointment date (i.e. when they still had supplies of metformin or placebo). Reported adherence was high, with 145/182 (79.7%) of participants in the metformin arm and 158/182 (86.8%) in the placebo arm indicating that they had taken >95% of their medicines in the last 28 days at their final assessment (*p*=0.068). Adherence was reportedly even higher among participants who had their final blood glucose tests within 6 days of their scheduled date (i.e. when they still had spare medicine): 98.2% (95% CI 97.4%, 99.1%) in the metformin arm (*n*=128) and 98.2% (95% CI 97.3%, 99.0%) in the placebo arm (*n*=149) (*p*=0.561).

**Table 2:** Rates of follow up and adherence to trial medications as reported by participants

Variable	Metformin	Placebo
Participants enroled, *n*	182	182
Follow-up duration (months), median (range)	12.0 (0.0–19.1)	12.0 (0.0–19.1)
Study completion and final 2h blood glucose testing status, *n*^a^		
Completed study: 48 week post glucose load data available or 24 week post glucose load ≥11.1 mmol/l	151	167
Did not complete study: 24 week post glucose load data available but no 48 week post glucose load data	2	3
Did not complete study: no 24 week or 48 week post glucose load data available	29	12
Timing of final glycaemia testing, *n* (%)		
As scheduled, at study end or within 6 days of final appointment	128 (70.3)	149 (81.9)
Between 7 and 29 days after the scheduled study end date (inclusive of the scheduled study end date)	11 (6.0)	9 (4.9)
On or after 30 days of their scheduled study-end visit	12 (6.6)	9 (4.9)
Did not complete the study	31 (17.0)	15 (8.2)
Proportion reporting >90% adherence to trial medications in the previous 28 days on the visual analogue adherence scale, *n*/*n* (%)^b^		
Week 2	153/170 (90.0)	167/176 (94.9)
Week 4	150/162 (92.6)	163/172 (94.8)
Week 12	141/154 (91.6)	161/168 (95.8)
Week 24	137/145 (94.5)	157/167 (94.0)
Week 36	122/135 (90.4)	147/158 (93.0)
Week 48	128/143 (89.5)	148/162 (91.4)

^a^Post glucose load defined as 2 h blood glucose following a 75 g glucose load

^b^Data from visual adherence score, which is based on the visual analogue scale (VAS) and used to assess the proportion of medication doses taken by the individual in the past month (visual adherence score ratings are between 0% and 100%)

Table 3 shows the outcome data at the study end. When comparing change in variables between baseline and 12 month readings, body weight reduced significantly over time in the metformin arm but not in the placebo arm. Changes in fasting glucose, glucose level 2 h following a 75 g glucose load and HbA_1c_ did not differ significantly by arm.

**Table 3:** Summary statistics and results from linear mixed models for primary and secondary outcomes with repeated measures and results from the generalised linear models for secondary outcomes without repeated measures

Outcomes	Metformin		Placebo		Mean difference (metformin−placebo)	
	*n*	Value	*n*	Value	Difference in least square means for metformin vs placebo (95% CI)	*p* value
Individuals enroled	182	–	182	–		
Data at 12 month follow-up, mean (95% CI)						
Fasting glucose, mmol/l	144	6.17 (6.03, 6.30)	162	6.30 (6.18, 6.42)	−0.08 (−0.37, 0.20)^a^	0.56
Glucose level 2 h post 75 g glucose load, mmol/l	144	7.88 (7.65, 8.12)	162	7.71 (7.49, 7.94)	0.20 (0.17, 0.58)^a^	0.28
HbA_1c_ (mmol/mol)	144	43.30 (41.08, 43.51)	162	43.51 (42.06, 44.96)	−1.44 (−3.58, 0.71)^a^	0.19
HbA_1c_ (%)	144	6.02 (5.91, 6.13)	162	6.13 (6.00, 6.26)	−0.13 (−0.33, 0.06)^a^	0.19
Weight, kg	144	75.71 (73.13, 78.28)	162	80.48 (77.52, 83.42)	−1.47 (−2.58, −0.35)^a^	0.01
LDL-C, mmol/l	143	2.98 (2.84, 3.13)	162	2.95 (2.81, 3.10)	0.10 (−0.06, 0.25)^b^	0.21
HDL-C, mmol/l	142	1.35 (1.29, 1.41)	162	1.31 (1.26, 1.37)	0.07 (0.00, 0.14)^b^	0.046
Total cholesterol, mmol/l	143	4.59 (4.44, 4.74)	162	4.58 (4.44, 4.72)	0.08 (−0.06, 0.22)^b^	0.25
Triglycerides, mmol/l	143	1.23 (1.12, 1.33)	162	1.17 (1.09, 1.26)	0.05 (−0.05, 0.16)^b^	0.30
Marginal mean (95% CI) change between baseline and 12 months						
Fasting glucose, mmol/l	144	−0.08 (−0.28, 0.13)	162	0.01 (−0.18, 0.21)	−0.08 (−0.37, 0.20)^a^	0.56
Glucose level 2 h post 75 g glucose load, mmol/l	144	−0.25 (−0.52, 0.02)	162	−0.46 (−0.71, −0.20)	0.20 (−0.17, 0.58)^a^	0.28
HbA_1c_, mmol/mol	143	3.56 (2.00, 5.12)	162	5.00 (3.53, 6.46)	−1.44 (−3.58, 0.71)^a^	0.19
HbA_1c_, %	143	0.33 (0.18, 0.47)	162	0.46 (0.32, 0.59)	−0.13 (−0.33, 0.06)^a^	0.19
Weight, kg	144	−2.18 (−2.98, −1.37)	162	−0.71 (−1.47, 0.05)	−1.47 (−2.58, −0.35)^a^	0.01

^a^Estimates derived using linear mixed models controlling for baseline values, including a participant level random intercept

^b^Estimates derived using linear regression models controlling for baseline values as lipids were only analysed at baseline and 12 month follow-up

HDL-C, HDL-cholesterol; LDL-C, LDL-cholesterol

When comparing variables between the metformin and placebo arms at study end, mean body weight was significantly lower in the metformin arm than in the placebo arm but mean fasting glucose, mean blood glucose levels 2 h post 75 g glucose load, and mean HbA_1c_ did not differ significantly between the two arms. Using a linear mixed model adjusting for baseline values, mean (95% CI) differences (metformin–placebo) were, −1.47 kg (−2.58, −0.35) for weight, −0.08 (−0.37, 0.20) for fasting glucose, 0.20 (−0.17, 0.58) for glucose levels 2 h post 75 g glucose load and −1.44 (−3.58, 0.71) for HbA_1c_ in mmol/mol (−0.13 [−0.33, 0.06] for HbA_1c_ in %). Findings were the same for covariate-adjusted models (data not shown). Furthermore, the mean levels of HDL-cholesterol, LDL-cholesterol, total cholesterol and triglycerides were not significantly different between the two arms at the study end.

The incident rate of participants reaching type 2 diabetes thresholds did not differ significantly between the study arms. In the metformin vs placebo arm incident rates per 100 person-years (95% CI) were 20.69 (13.63, 30.10) vs 24.93 (17.36, 34.67) for a fasting glucose value of 7.0 mmol/l or higher (*p*=0.335), 7.71 (3.98, 13.46) vs 5.25 (2.40, 9.96) for blood glucose levels 2 h post glucose load of 11.1 mmol/l or higher (*p*=0.514) and 29.31 (20.42, 40.77) vs 37.68 (28.14, 49.41) for an HbA_1c_ of 48 mmol/mol (6.5%) or more (*p*=0.147).

Table 4 shows safety data. The frequencies of Grade 3 and Grade 4 events were similar between the two arms. Overall 16/182 individuals (8.8%) had a Grade 3 or Grade 4 event or died in the metformin arm compared with 18/182 individuals (9.9%) in the placebo arm. All of these events were classed as either being unrelated to or unlikely to be related to the study drugs. Grade 3 or Grade 4 adverse events were present at baseline in 9/16 participants (56.3%) who had Grade 3 or Grade 4 adverse event or died in the metformin arm of the trial, compared with 9/18 individuals (50.0%) in the placebo arm.

**Table 4:** Distribution of adverse events over 12 months of follow up in the metformin and placebo arms, stratified by severity

Variable	Metformin	Placebo
Participants at baseline, *n*	182	182
Participants with Grade 3 or Grade 4 adverse events during follow up, *n*		
Deaths	1	2
Participants with any Grade 4 event	1	2
Participants with any Grade 3 event	15	14
Participants with any Grade 3 or Grade 4 event or who died	16	18
Participants with any Grade 3 or Grade 4 event at baseline who had a Grade 3 or Grade 4 event or who died during follow up	9	9
Participants seen after baseline visit, *n*	174	177
Any abnormal liver function-associated events post baseline at Grade 3 or Grade 4, number of events	2	2
Any abnormal lipid profile events post baseline at Grade 3 or Grade 4, number of events	2	0
Any abnormal renal function events post baseline at Grade 3 or Grade 4, number of events	1	0
Any abnormal FBC results post baseline at Grade 3 or Grade 4, number of events^a^	2	2
Hospitalisations/other at Grade 3 or Grade 4 after baseline, number of events	1	3
Grade 1 or Grade 2 symptoms, number of events post baseline		
≥1 Grade 1 or Grade 2 symptom	118	96
Diarrhoea	60	17
Vomiting	26	7
Abdominal pain	21	16
Nausea	54	29
Loss of appetite	27	17
Flatulence	25	17
Fatigue	25	27
Headaches	18	26
Dizziness	20	13
Other	50	47
Any Grade 1 or Grade 2 symptoms at different follow-up times, number of events/number of participants		
2 week visit^b^	107/170	76/175
1 month visit^b^	43/162	26/172
3 month visit	28/155	22/169
6 month visit^c^	6/144	6/166
9 month visit^d^	1/134	3/156
12 month visit^e^	2/140	4/156

Adverse event grading is based on the DAIDS grading scale: Grade 3 indicates a severe event; Grade 4 indicates a potentially life-threatening event

^a^FBC data only available at baseline and 12 month follow-up

^b^Missing data: *n*=0, metformin arm; *n*=1, placebo arm

^c^Missing data: *n*=1, metformin arm; *n*=1, placebo arm

^d^Missing data: *n*=1, metformin arm; *n*=2, placebo arm

^e^Missing data: *n*=4, metformin arm; *n*=6, placebo arm

Two people in the metformin arm had multiple adverse events: one had two Grade 3 adverse events (suspected cervical carcinoma and HIV-related raised liver enzymes), and the other had three Grade 3 adverse events (liver insufficiency secondary to HIV or hepatitis, hypoalbuminaemia secondary to malnutrition, and severe anaemia secondary to malnutrition and chronic infection) and then died. No individuals had multiple adverse events in the placebo arm. The were no cases of lactic acidosis.

Grade 1 and Grade 2 symptoms, in particular diarrhoea, vomiting and nausea, were reported more frequently in the metformin arm than in the control arm. Overall, there were 118 individuals with Grade 1 or Grade 2 symptoms in the metformin arm over the 12 months and 96 individuals in the placebo arm (IRR: 1.35 (95% CI 1.03, 1.79); *p*=0.0273). Of all participants seen after the baseline visit, 56/174 individuals (32.2%) in the metformin arm and 81/177 (45.8%) in the placebo arm did not report any Grade 1 or Grade 2 events (*p*=0.009, assessed by *χ*^2^ test). Grade 1 or Grade 2 symptom reporting was higher in the metformin arm at the 2 week and 1 month visits (*p*<0.001 and *p*=0.010, respectively, assessed by *χ*^2^ test). From the 3 month visit onwards, all differences in Grade 1 or Grade 2 symptom reporting were non-significant (Table 4).

## Discussion

In this Phase II double-blind placebo-controlled trial among people with HIV and prediabetes, there was no significant difference in glucose levels between the metformin and placebo arms at the study end when measured by glucose levels 2 h after a 75 g glucose load or by fasting glucose. However, glucose levels 2 h post glucose load fell in both arms, and fasting blood glucose was lower at study end than at enrolment in the metformin arm but not in the placebo arm, although these declines were not significant when we controlled for baseline values.

Whilst assessment of safety was not the primary endpoint of the trial, none of the abnormal laboratory/biochemical results or clinical adverse events that we observed at Grade 3 or higher could be linked to metformin. We observed no cases of lactic acidosis, an extremely rare and serious complication that has been linked to being on ART and metformin [41–43].

Why did the trial show no differential effect of metformin on blood glucose compared with placebo at the 12 month follow-up? It is possible that, in this population, metformin is either ineffective or that the effect was too small for the study to detect (i.e. the study was underpowered). Our study population was comprised of mostly women who were not pregnant and this distribution was a weakness. Metformin did not confer protection against progression to type 2 diabetes among women who did not have a history of gestational diabetes in a large study in the USA that had a 10 year follow-up [9]. The number of participants that we recruited with blood glucose levels close to the type 2 diabetes threshold and who were obese, who may have benefited most from metformin [6, 12, 44], was small. Also, our follow-up period was just 12 months, compared with 30 months or more in the United States Diabetes Prevention Programme [6] or the Indian Diabetes Prevention Programme [18]. Thus, it is possible that the short duration of follow up in our trial had an impact on the assessment of efficacy of metformin with regard to blood glucose levels, with the effects of metformin potentially taking longer to establish.

Why glucose levels 2 h post glucose load fell in both arms is not clear. Metformin reduces energy intake, attenuates weight gain and, thus has a beneficial effect on body weight and visceral fat [45]. We saw a substantive effect on body weight in the metformin arm and not in the placebo arm. This is in line with what has been observed in other studies, which have demonstrated an effect of metformin on type 2 diabetes incidence in larger sample sizes followed over a longer duration [11, 12]. Finally, we also saw a decline in fasting glucose over the 12 months in the metformin group and not in the placebo group, although this was non-significant. Metformin suppresses endogenous glucose production, a determinant of fasting plasma glucose concentrations, which is in line with this finding [6, 46–48]. As over 90% of participants in both arms were on dolutegravir-based regimens, there did not appear to be any clinical indication of an attenuating effect of dolutegravir on metformin action.

We had severe disruption from COVID-19 whilst conducting the study. The COVID-19 pandemic started shortly after the trial began and government bodies and media started giving regular advice on the importance of a healthy diet and exercise to protect health from COVID-19, particularly for those living with type 2 diabetes. We also provided participants with basic diet and lifestyle advice at each contact, as planned. However, our contact with participants increased because of the disruption caused to health services by COVID-19 and so the frequency of advice that participants received also increased. We know from studies conducted in high-income settings that intensive structured promotion of diet and exercise is more powerful than metformin in reducing type 2 diabetes risk and that metformin has little added effect if there is substantial behaviour change related to diet and exercise [6, 20]. Our participants did not receive an intensive structured behavioural change intervention but in the setting in SSA, where access to information on diet and exercise is limited, provision of limited information combined with the fear of COVID-19 may have had an effect on diet and lifestyle behaviour, thus attenuating the effect of metformin.

It is evident from the changes in weight (and also lipid markers [data not shown]), which improved in both arms, that diet and lifestyle behaviour changed. Metformin’s ability to improve lipid makers among individuals without diabetes but with HIV has been shown by others [49].

Pharmacological interventions can be implemented with relative ease in our population, giving them alongside ART. Diet and exercise interventions, as tested in high-income settings, are time intensive and would be costly to implement in SSA. A recent randomised trial from South Africa comprising about 500 participants who were followed for 7–9 months, demonstrated that a behavioural intervention based on video group sessions was feasible and lowered HbA_1c_ but had no effect on weight, BP or triglyceride levels [50]. Our trial and these findings from South Africa suggest that behavioural interventions for the reduction of type 2 diabetes incidence should be explored further.

Of note, despite the severe disruption caused by the COVID-19 pandemic, over 70% of participants attended as scheduled throughout the year. This was largely because of the time taken by study researchers to build trust and partnership with participants. We did not titrate the dose of metformin, as is commonly done in the management of type 2 diabetes, primarily for ease of administration and because we felt that the extended-release formulation would be well tolerated. Despite this and the comparatively higher frequency of Grade 1 and Grade 2 side effects in the metformin arm compared with the placebo arm, reported adherence to medication was very high in both arms, and was over 98% among those who attended clinic appointments as scheduled.

Our study demonstrates the high frequency of IFG and/or IGT that is present, and which remains largely undetected, in people living with HIV in SSA. It also demonstrates that it is feasible to integrate diabetes services within HIV programmes. These findings support other recent research studies conducted in this setting [51, 52]. Health policies are now recommending integrated management of chronic conditions in SSA [53].

In SSA, type 2 diabetes affects people at a younger age than in high-income countries [1, 54] and is having a massive impact on the continent. Evaluating interventions for the prevention of type 2 diabetes in high-risk groups, such as those with HIV and concomitant prediabetes, is critical. This early-phase trial shows that metformin was safe in our study cohort but its effect on glycaemia was unclear. A larger study with a longer follow up is needed to test the efficacy of metformin in delaying or preventing type 2 diabetes onset in individuals with HIV and prediabetes.

## Data Availability

The data that support the findings of this study are not openly available due to reasons of sensitivity and are available from the corresponding author upon reasonable request and with the permission of the RESPOND-Africa partnership.

## Appendix

### Additional members of the META trial team

**Muhimbili Medical Research Centre, National Institute for Medical Research, Dar es Salaam, Tanzania:** Fredrick Amani, Bilton Exavery, Ally Ibrahim, Calvin Kaaya (Pharmacist), Magdalena Kiondo, Emiliana Kokwitangilira, Neema Lyasenga, Harrieth Manisha, Loycea Massanja, Tumaini Mlagha, Neema Moshi, Shaany Mwaruka, Rehema Mzarau, Bora Nchimbi, Jackline Peter, Francisca Pratap, Asia Ramadhani (Pharmacist), Elibariki Raymond, Emmanuel Sareya, Sezari Sezanye, Diana Traufoo, Tom Magehema (Pharmacist).

**Muhimbili National Hospital, Dar es Salaam, Tanzania:** Deus Burma (Chief Pharmacist), James Kalabashanga, Justace Bwire, Idda Tarimo (Pharmacist).

**Shree Hindu Mandal Hospital, Dar es Salaam, Tanzania:** Zainabbas Ladha (Pharmacist), Stephen Muya.

**Department of International Public Health, Liverpool School of Tropical Medicine (LSTM), Liverpool, UK:** Caroline Jeffries, Faith Moyo, Louis Niessen, Joseph Okebe, Hazel Snell, Jonathan Willitts.

**Steering Committee:** Professor Peter Smith (Chair), MRC International Statistics and Epidemiology Group, London School of Hygiene and Tropical Medicine (LSHTM), London, UK; Professor Nelson Sewankambo, School of Medicine, Makerere University College of Heath Sciences, Kampala, Uganda; Dr Josephine Birungi, MRC/UVRI/LSHTM Uganda Research Unit, Entebbe, Uganda; Dr Janet Lutale, Muhimbili University of Health and Allied Sciences, Dar es Salaam, Tanzania; Professor Geoff Gill, Emeritus Professor, Liverpool School of Tropical Medicine (LSTM) and University of Liverpool, Liverpool, UK.

**Data Safety Monitoring Committee:** Professor John Wilding (Chair), Department of Cardiovascular and Metabolic Medicine, Aintree University Hospital, Liverpool, UK; Dr David Macleod, MRC International Statistics and Epidemiology Group, London School of Hygiene and Tropical Medicine (LSHTM), London, UK; Professor Thomas Harrison, St Georges Hospital, University of London, London, UK; Professor Andrew Kitua, Public Health and Environment Advancement Interventions NGO (NGALAKERI), Morogoro, Tanzania.

## Abbreviations

ART: Antiretroviral therapy
COVID-19: Coronavirus disease 2019
DAIDS: Division of Acquired Immunodeficiency Syndrome
FBC: Full blood count
IFG: Impaired fasting glucose
IGT: Impaired glucose tolerance
IRR: Incidence rate ratio
LSTM: Liverpool School of Tropical Medicine
SAE: Serious adverse event
SSA: Sub-Saharan Africa
